# Aldosterone antagonist therapy and its relationship with inflammation, fibrosis, thrombosis, mineral-bone disorder and cardiovascular complications in peritoneal dialysis (PD) patients

**DOI:** 10.1007/s11255-017-1655-2

**Published:** 2017-07-14

**Authors:** Rafał Donderski, Paweł Stróżecki, Beata Sulikowska, Magdalena Grajewska, Ilona Miśkowiec, Anna Stefańska, Joanna Siódmiak, Grażyna Odrowąż-Sypniewska, Jacek Manitius

**Affiliations:** 1 Department of Nephrology, Hypertension and Internal Medicine, Collegium Medicum in Bydgoszcz, Nicolaus Copernicus University Toruń, Toruń, Poland; 2 Department of Clinical Laboratory Medicine, Collegium Medicum in Bydgoszcz, Nicolaus Copernicus University Toruń, Toruń, Poland; 3 Skłodowskiej-Curie No 9 Street, 85-094 Bydgoszcz, Poland

**Keywords:** Peritoneal dialysis, Inflammation, Fibrosis, Thrombosis markers, Mineral and bone markers, Cardiovascular complications

## Abstract

**Background:**

High aldosterone level may contribute to pathogenesis of hypertension, vessels damage and cardiovascular system deterioration in chronic kidney disease patients. Besides its classical action via mineralocorticoid receptor, aldosterone is also involved in cell growth, inflammation, oxidative stress, endothelial dysfunction and exerts fibroproliferative effects. The aim of the study was to assess whether aldosterone antagonist treatment may influence serum level of inflammatory, fibrosis, thrombosis and mineral-bone metabolism markers in peritoneal dialysis (PD) patients and blood pressure, aortic stiffness, echocardiographic indices after 12 months of treatment.

**Methods:**

Twenty-two patients on PD were assigned to spironolactone treatment in dose of 50 mg daily during 12 months. Fifteen PD patients were assigned to control group. Echocardiographic indices, PVW, SBP, DBP (mean values from ABPM) and biochemical parameters such as: aldosterone, osteopontin, IL-6, selectin-P, TGF-β, PTH, MMP-2 were performed at the beginning and after 12 months in spironolactone and control group.

**Results:**

There were no statistically significant differences in echocardiographic indices, PWV, BP (ABPM readings) and biochemical markers: MMP-2, serum aldosterone, TGF-β, IL-6, selectin-P, PTH level after 12 months of spironolactone treatment. There was statistically significant rise in osteopontin level after 12 months of spironolactone treatment. Episodes of life-threatening hyperkalemia were not reported.

**Conclusions:**

Aldosterone antagonists use in PD patients seems to be safe. Longer duration or higher dosage of spironolactone seems to be more effective in improving cardiovascular system status in PD patients. Further studies are required to determine relationship between mineralocorticoid receptor blockade and mineral-bone disturbances in PD patients.

## Introduction

Activation of renin-angiotensin-aldosterone system (RAAS) plays crucial role in pathogenesis of essential hypertension, hypertension related with kidney diseases and congestive heart failure (CHF). There is a lot of evidence that excess of aldosterone is an important risk factor of cardiac hypertrophy and fibrosis, subclinical inflammation, endothelial dysfunction, tissue fibrosis and vessels obliteration. Moreover, persistent hyperaldosteronemia plays an important role in progression of chronic kidney disease (CKD) [1]. Based on Rales Study and Ephesus Study, aldosterone antagonist is often complemented to angiotensin converting enzyme inhibitors (ACE-Is) or antagonists of angiotensin I (ARBs) treatment of congestive heart failure (CHF) and cardiac failure related to myocardial infaction [2]. Mineralocorticoid receptor (MR) antagonists improve the survival in CHF and are widely recommended in this condition [2]. Mineral and bone disorder (MBD) in CKD patients (CKD-MBD), chronic inflammation, endothelial dysfunction, tissue fibrosis are common finding in these patients and may be related with progression of cardiovascular disease in this population. Aldosterone seems to play an important role in bone remodeling in CKD patients. Excess of aldosterone is responsible for calcium and magnesium urine and digestive tract excretion and depletion of these ions in serum. Excess of aldosterone is responsible for increase of parathormone (PTH) and decrease of mineral-bone density and bones strength [3]. Aldosterone antagonist is rarely prescribed in CKD patients especially on conservative treatment because of risk of severe hyperkaliemia. In opposite to these limitations, these agents are more frequently prescribed in dialysis patients, and this therapy is considered to be safe. In hemodialysis patients such treatment is possible but requires strict potassium control [4]. It has been pointed out recently that in hemodialysis patients spironolactone treatment (in a dose 25 mg daily) reduces high incidence of cardiovascular and cerebrovascular mortality after 3-year treatment [5]. The aim of the study was to assess the influence of 12 months of spironolactone treatment on inflammatory, fibrosis, thrombosis and mineral-bone markers and cardiovascular system status (echocardiographic indices, arterial stiffness, BP values) in PD patients.

## Patients and methods

Thirty-seven Caucasian patients (pts) were examined: 21 female (F) and 16 male (M), mean age 52, 85 ± 15.8 years (median: 51 years; range 20–76 years). They were recruited from PD center of Department of Nephrology, Hypertension and Internal Medicine in University Hospital in Bydgoszcz. All patients were on conventional automated peritoneal dialysis (APD) using Home Choice or Home Choice Pro devices delivered by Baxter. The causes of end-stage renal disease (ESRD) were: diabetic kidney disease—20 pts (type 1 diabetes—3 pts, type 2 diabetes—17 pts), non-diabetic nephropathies—17 pts. The latter group included as follows: chronic glomerulonephritis (CGN)—10 pts; obstructive nephropathy—2 pts; hypertensive nephropathy—5 pt. Comorbid conditions at the onset of the study were: coronary artery disease was diagnosed in 15 pts, symptoms of CHF were present in 10 pts. All of the patients enrolled into the study suffered from hypertension, and they required antihypertensive medication (2 or more antihypertensive agents). Treatment of hypertension was in accordance with current guidelines of ESH/ESC. The goal of treatment was to reach blood pressure below <140/90 mmHg. The following medications were prescribed: ACE-Is—29 pts, ARBs—8 pts, calcium channel blockers—26; Beta-blockers—15 pts, loop diuretics—30 pts with residual renal function. Chronic kidney disease–mineral and bone disorder (CKD-MBD) were treated according to KDIGO guidelines 2016 (update on the management of CKD-MBD) using conventional therapy: vitamin D (alfacalcidol 3 times a week in a dose from 0.25 to 0.5 µg daily) and calcium–phosphorus binders (calcium carbonate prescribed daily dose from 3.0 g to 9.0 g). There was no need to use calcimimetics. At the beginning of the study patients were divided into 2 groups. Twenty-two patients were treated with spironolactone orally in the dose of 50 mg daily during 12 months. Fifteen patients who refused spironolactone treatment as an additional medication (they were on traditional treatment including ACE-I or ARBs) served as a control group. There were 12 diabetic patients in spironolactone group and 8 diabetic in control group. Inclusion criteria were: PD treatment time >6 months, stable clinical condition 6 weeks prior to enrollment without any inflammation, age >18 years old, written informed consent, no data of immunological disease with need for immunosuppressive treatment. Patients were eligible for spironolactone group when mean serum potassium level after 3 ambulatory visits was <5.0 mmo/l. Twenty-four-hour ambulatory blood pressure monitoring (ABPM) using A&D TM 2430 device was performed in each patient at the onset of the study and after 12 months. Echocardiographic evaluation (LVMI), arterial stiffness with assessment of carotid-femoral pulse wave velocity (PVW) using the Complior^®^ device (Artech Medical, Pantin, France) were performed in each individuals at the onset and after 12 months of the study. Blood samples for serum aldosterone level [pg/ml], metalloproteinase-2 (MMP-2), osteopontin (OPN), transforming growth factor-beta (TGF-β), P-selectin, interleukin-6 (IL-6) were taken in each subject at the commencement and after 12 months of the study. Serum PTH was measured using a chemiluminescent immunoassay on the DiaSorin LIAISON. Serum aldosterone, osteopontin, IL-6, TGF beta, MMP-2, P-selectin were determined by a sandwich ELISA method. Serum Aldosterone using IBL International ELISA. Osteopontin, IL-6, TGF beta, MMP-2 and P-selectin using R&D Systems ELISA. During the observation period, patients were on conventional APD (Baxter system) using 1.36%/2.27% glucose solution (Dianeal, Baxter) with 5–6 overnight exchanges. The conventional APD schedule was: single dwell volume: 2.0–2.3 L, dwell time was—1.0 h and 30 min, the total dialysis time was about 10–11 h. We do not use Physionel and Nutrineal. Extraneal was used in 10 pts with overhydration signs. During the observation period we routinely carried out dialysis adequacy test (assessment of kt/v index) and peritoneal permeability tests (PET). Residual renal function (RRF) was present in all individuals, 10 pts with RRF decline and daily diuresis <500 ml was reported. During the observation period, there were no dropouts, transfers to HD related to either technique failure or infectious complications such as peritonitis or exit site infection. Clinical characteristics of the patients are shown in Table 1. The study protocol was approved by the local Bioethics Committee on Nicolaus Copernicus University in Toruń.

**Table 1:** Clinical characteristics of the patients at the beginning of the study

Parameter	Spironolactone group (*n* = 22)	Control group (*n* = 15)	*p*
Age (years)	54.57 ± 13.78	51.14 ± 18.72	0.70
BMI (kg/m^2^)	25.16 ± 3.58	24.05 ± 4.42	0.61
Time on PD at the onset of the study (months)	25.28 ± 9.46	20.42 ± 10.69	0.38
Hemoglobin concentration (g/dl)	11.45 ± 0.97	11.55 ± 1.39	0.65
Serum calcium (mmol/l)	2.29 ± 0.24	2.28 ± 0.13	0.28
Serum phosphorus (mmol/l)	1.60 ± 0.30	1.75 ± 0.58	0.55
PTH (pg/ml)	306.00 (125.0;759.0)	376.00 (98.0;805.0)	0.22
Serum albumin (g/dl)	4.17 ± 0.23	3.99 ± 0.20	0.60
Urine volume (ml/day)	914.28 ± 94.49	878.57 ± 191.17	0.66
UF volume (ml/day)	872.85 ± 96.55	910 ± 134.90	0.56
PET			
H	7	5	0.14
HA	12	8	0.34
LA	3	2	0.54
L	0	0	

Normally distributed data are expressed as mean ± SD, and not normally distributed data are expressed as median ± IQR

*BMI* body mass index, *PET* peritoneal equilibration test, *UF* ultrafiltration, *PTH* parathormone, *PD* peritoneal dialysis; *H* high, *HA* high average, *LA* low average, *L* low

### Statistical analysis

Statistical analysis was performed using the Statistica 7.0 PL software (StatSoft Inc., Tulsa, OK, USA). The obtained data are presented as mean ± standard deviation (SD), and the median and top and bottom quartiles are given for variables that were not normally distributed. Distribution of variables was analyzed using the Shapiro–Wilk test. Statistical analysis was performed using the Student’s *t* test. If any variable was not normally distributed, the *U* Mann–Whitney test was used. Qualitative data were compared by means of the *χ* ^2^-test. Linear correlation between variables was analyzed using: Spearman rank correlation coefficient (for samples with non-normal distribution) and Pearson’s correlation coefficient (for samples with normal distribution) also. *P* value < 0.05 was considered as statistically significant.

## Results

Mean values and median values of investigated parameters at the onset and after 12 months of the study in spironolactone and control group are shown in Table 2. Clinical characteristics of patients at the study onset are presented in Table 1. There were no changes in cardiovascular system status in spironolactone treatment group and control group after 12 months of study. There was statistically significant linear correlation in spironolactone treatment group between IL-6 versus MMP-2 (*r* = −0.91; *p* < 0.05) at the onset of the study. Statistically significant correlation between TGF-β versus aldosterone (*r* = 0.77; *p* < 0.05) was found in control group after 12 months of observation. Moreover, significant correlation was found between ΔOPN versus ΔTGF-β (*r* = 0.76; *p* < 0.05), ΔP-selectin versus ΔTGF-β (*r* = 0.80; *p* < 0.05) and ΔPWV versus ΔOPN (*r* = 0.76; *p* < 0.05) in spironolactone treatment group. These data are presented in Table 3. Peritoneal membrane type after 12 months of observation was as follows—spironolactone treatment group (number of pts): H-9; HA-10; LA-3; L-0. Control group (number of pts.): H-5; HA-8; LA-2; L-0. Initial assessment data are presented in Table 1.

**Table 2:** Characteristics of the studied group of PD patients (*n* = 22) during 12 months of spironolactone treatment and characteristics of the control group (*n* = 15) during 12 months of observation

Parameter	Treatment group (*n* = 22)		P 1 vs 2	Control group (*n* = 15)		P 3 vs 4
	Baseline (1)	After 12 months (2)		Baseline (3)	After 12 months (4)	
ALD (pg/ml)	289.90 (114.0;466.50)	215.20 (176.7;371.3)	0.86	108.50 (53.70;189.90)	181.70 (55.10;317.00)	1.00
MMP-2 (ng/ml)	257.90 (256.1;328.9)	252.30 (217.4;312.1)	0.39	311.60 (251.00;386.20)	333.10 (287.20;437.10)	0.44
OPN (ng/ml)	252.30 (45.5;109.8)	209.40 (110.6;264)	0.01	42.50 (30.30;149.00)	90.40 (70.90;600.00)	0.09
TGF-β (ng/ml)	35.8 ± 10.4	30.6 ± 6.8	0.87	33.8 ± 9.7	29.6 ± 5.1	0.38
P-selectin (ng/ml)	126.00 (84.0;265.0)	147.00 (76.0;242.0)	0.86	155.00 (95.00;271.00)	98.00 (49.00;132.00)	0.13
IL-6 (pg/ml)	4.7 ± 2.7	7.1 ± 5.6	0.33	6.8 ± 7.4	4.8 ± 3.8	0.49
Ca (mmo/l)	2.29 ± 0.24	2.18 ± 0.25	0.45	2.28 ± 0.13	2.34 ± 0.24	0.61
P (mmo/l)	1.60 ± 0.30	1.55 ± 0.24	0.72	1.75 ± 0.58	2.00 ± 0.58	0.43
PTH (pg/ml)	306.00 (125.0;892.0)	252.00 (86.0;535.00)	0.14	376.00 (98.0;965.0)	297.00 (112.0;859.0)	0.60
K (mmol/l)	4.87 ± 0.37	5.02 ± 0.22	0.36	4.75 ± 0.39	4.89 ± 0.91	0.41
24-h SBP (mmHg)	142.14 ± 15.23	135.12 ± 18.56	0.76	133.57 ± 30.64	139.12 ± 13.7	0.45
24-h DBP (mmHg)	87 ± 10.40	80 ± 12.78	0.54	82.85 ± 22.88	86.50 ± 34.12	0.59
PWV (m/s)	11.51 ± 5.29	11.92 ± 3.64	0.82	10.11 ± 6.29	11.05 ± 3.28	0.68
LVMI (g/m^2^)	122.17 ± 24.32	139.58 ± 21.41	0.18	125.77 ± 24.32	129.12 ± 18.36	0.27
Kt/v index	1.93 ± 0.17	2.13 ± 0.17	0.06	1.98 ± 0.26	2.01 ± 0.40	0.85
Urine volume (ml/day)	914.28 ± 94.49	821.42 ± 56.69	0.05	878.57 ± 191.17	771.42 ± 149.60	0.26
UF volume (ml/day)	872.85 ± 96.55	817.14 ± 89.76	0.28	910 ± 134	885.71 ± 140.57	0.74

Data are expressed as mean ± SD and median plus interquartile range (IQR)

*ALD* serum aldosterone level, *MMP-2* metalloproteinase-2, *TGF-β* transforming growth factor-β, *IL-6* interleukin-6, *PTH* parathormone, *PWV* pulse wave velocity, *LVMI* left ventricular mass index, *24-h SBP* 24-h systolic blood pressure, *24-h DBP* 24-h diastolic blood pressure

**Table 3:** Correlation coefficients between ΔOPN (OPN12-OPN0), ΔTGF-β(TGFβ12-TGFβ0), ΔP-selectin (P-selectin12-P-selectin0) in spironolactone treatment group, and selected parameters

Parameter	ΔOPN		ΔTGF beta		ΔP-selectin	
	*r*	*R*	*r*	*R*	*r*	*R*
ΔALDO	0.29	0.54	0.11	0.36	0.42	0.57
	*p* = 0.521	*p* = 0.215	*p* = 0.815	*p* = 0.431	*p* = 0.353	*p* = 0.180
ΔMMP-2	0.32	0.04	−0.24	−0.32	−0.20	0.04
	*p* = 0.490	*p* = 0.939	*p* = 0.602	*p* = 0.482	*p* = 0.667	*p* = 0.939
ΔOPN	1.00	–	*0*.*76*	*0*.*86*	0.53	0.29
	–	–	*p* = *0*.*046*	*p* = *0*.*013*	*p* = 0.221	*p* = 0.534
ΔTGF-β	*0*.*76*	*0*.*86*	1.00	–	*0*.*80*	0.50
	*p* = *0*.*046*	*p* = *0*.*013*	–	–	*p* = *0*.*031*	*p* = 0.253
ΔP-selectin	0.53	0.29	*0*.*80*	0.50	1.00	–
	*p* = 0.221	*p* = 0.534	*p* = *0*.*031*	*p* = 0.25	–	–
ΔIL-6	−0.19	−0.07	0.13	0.39	0.09	0.18
	*p* = 0.688	*p* = 0.879	*p* = 0.782	*p* = 0.383	*p* = 0.849	*p* = 0.701

*r* Pearson’ coefficient, *R* Spearman rank coefficient

Italic values denote statistical significant (*p* < 0.05)

## Discussion

Cardiovascular complications are major cause of increased mortality in dialysis patients and tend to aggravate in the course of dialysis treatment. Increased aldosterone level is considered to be one of the non-traditional cardiovascular risk factors which may be responsible for unsuccessful prognosis in PD patients. Hyperphosphatemia, increased PTH level, inflammation, endothelial dysfunction, oxidative stress are also common non-traditional markers of high CV risk in dialysis patients [6]. Aldosterone is a pleiotropic hormone which activates mineralocorticoid receptors (MR) in different epithelial and non-epithelial tissues. The effects of excess of aldosterone are related with enhanced collagen type I synthesis, endothelial dysfunction (inhibition of iNOS activity and impairment of vasodilatation), initiation of inflammation or disturbances in hemostasis system. Another place of aldosterone action via mineralocorticoid receptors (MR) is bones. MR are present on osteoblasts. The literature data concerning aldosterone-induced effects on bones metabolism or role of MR blockade especially in aspect of CKD-MBD are rarely investigated. All mentioned actions facilitate cardiovascular damage [7]. In our study an interventional group of 22 patients was treated with 50 mg spironolactone daily. We observed a trend for serum aldosterone level decline after 12 months (without statistical significance). There were not any statistically significant changes of the markers of inflammation, fibrosis, thrombosis such as interleukin 6 (IL-6), TGF-β, P-selectin or MMP-2. We suppose that the dose of spironolactone was too small or the time of treatment was too short to make any changes in these parameters. There was only statistically significant rise in serum osteopontin level after 12 months of spironolactone treatment. Osteopontin is considered to be marker of acute and chronic inflammation, neoangiogenesis, carcinogenesis (high level of osteopontin is detected in solid tumors and hematological malignances). Osteopontin is also regarded as a potent inhibitor of vessels calcification [8, 9]. Moreover, there is a strong evidence that osteopontin may be considered as a one of a non-traditional cardiovascular risk factor related with poor prognosis in CKD patients [10]. Increased osteopontin and osteocalcin levels correlated with rise of PTH level in PD patients may suggest an enhancement of bone turnover and calcification process [11]. In our study, we reported a tendency to diminish initial PTH level in spironolactone group after 12 months of therapy (without statistical significance). We can only speculate if it is an effect of traditional CKD-MBD treatment or spironolactone additional properties. High level of osteopontin after 12 months in our study may be a result of persistent hyperglycemia related with standard glucose fluids or chronic subclinical inflammation related with bioincompatible peritoneal fluids. But we are aware that such explanation is not good enough, because same PD fluid was prescribed in control group. Inhibition of MR expressed on osteoblasts and osteocytes due to spironolactone may result in stimulation of bone formation and suppression of resorption. Bidirectional interactions between aldosterone and parathyroid hormone are raised in the literature and may have potential effects on the cardiovascular system and bone metabolism [12]. Probably, spironolactone administration may enhance osteopontin release to inhibit calcification process. We may speculate that, lowering PTH level in renal patients is not only related with conventional treatment but may be partially related with MR inhibition. In our opinion rise of osteopontin during spironolactone treatment may be valuable to inhibit calcification process, on the other hand we may speculate that it is only a consequences of increased calcium turnover and advanced calcification process in our patients [13]. In conducted study we find positive correlation between ΔOPN and TGF-β and ΔP-selectin that may indicate close relationship between these risk markers which facilitate cardiovascular system damage. (Table 3)

In opposite to other investigators reports, there were not any significant differences in blood pressure, LVMI, PVW in spironolactone group at the baseline versus after 12 months (only a tendency in SBP/DPB decrease after 12 months of treatment without statistically significance). Data concerning the influence of spironolactone or eplerenone treatment on ameliorating cardiovascular damage in PD patients are sparse. Most trials were performed in hemodialysis patients [14]. Ito and co-workers in their study tried to evaluate long-term effects of spironolactone treatment in PD patients. They conducted multicenter, prospective, randomized trial of 158 patients undergoing PD therapy and receiving either traditional treatment—ACE-Is or ARBs (control group) versus combined treatment with spironolactone for 2 years. The routine dose of spironolactone in their study was 25 mg daily. Echocardiographic evaluation disclosed significantly diminished LVMI in the spironolactone group at 6, 18, 24 months of treatment in compare to control group. Moreover, it was significant improvement in ejection fraction (EF) in spironolactone group versus control group after 24 months of treatment. On the contrary, in control group significantly higher LVMI was reported after 24 months in compare to the baseline. Unfortunately, authors of the study were unable to prove any favorable effects of active spironolactone treatment on residual renal function (urine volume), changes of kt/v or peritoneal membrane function assessed by D/P for creatinine [15]. Hausmman and Cohen described a successful treatment using 25 mg aldactone daily in diabetic nephropathy patient with CHF on PD. The small dose of aldactone added to traditional treatment resulted in improvement of ejection fraction (EF) from 32 to 46%, 10 months after this therapy had been initiated. The serum potassium level did not exceed 5.5 mmo/l during the period of observation [16]. Favorable effects of 6-month spironolactone administration in CAPD patients with CHF (NYHA class III and IV) and significant rise in EF were also reported by Taheri et al. [17]. In our own study we were unable to show the statistically significant changes in blood pressure after commencing spironolactone administration. We observed only a trend toward lowering BP without statistical significance. There were no changes in PVW after 12 months of treatment. Stróżecki and colleagues reported elevated PVW in PD patients in compare to healthy control. PVW was significantly higher in diabetic than non-diabetic PD patients. Increased PVW in PD patients was associated with age, diabetic status and longer duration of PD therapy but not with the type of peritoneal membrane transport [18]. In our study we reported only tendency to higher PWV in diabetic spironolactone treatment group in compare to non-diabetic (without statically significance). Vukusich et co-workers in hemodialysis patients who received spironolactone trice weekly in a dose 50 mg daily observed decrease or even reversed carotid intima media thickness after 2 years of treatment [19]. The dose of spironolactone that we advised to our patients was 50 mg daily, and it seems to be even higher in compare to 25 mg daily in former trials. It is hard to explain why this dose was insufficient in our patients. During our observation, we did not detect the problem of hyperkalemia related with spironolactone treatment. Because of lack of potassium in conventional peritoneal fluids and continuous nature of this form of renal replacement therapy, hyperkalemia is rather rare complication of such treatment. In our study spironolactone therapy seems to have no influence on RRF, residual diuresis decline, dialysis adequacy or peritoneal membrane permeability in PET test. (These data are shown in “Results” section and Table 2). The lack of clearly seen spironolactone influence on investigated parameters as we mentioned before may be explained by short-term treatment, too short to exert any changes. It is hard to establish and recommend an optimal time of spironolactone treatment to report favorable changes. Besides of our own results, according to other studies, MR antagonists seem to be a valuable therapeutical option in PD patients [15]. Another clinical problem that requires solution is peritoneal membrane destruction after a few years of dialysis. So far, we have only a few studies with description of favorable effects of ACE-I on peritoneal membrane integrity [20]. Aldosterone receptor blockers seem to be another valuable agent to inhibit peritoneal membrane sclerosis and calcification. The limitation of the study was too short observation period and relatively small number of patients that can limit statistical analysis. Another limitation was common ACE-I and ARBs use in hypertension or congestive heart failure treatment with its influence on renin-angiotensin-aldosterone axis inhibition. Moreover, we were unable to assess all mentioned proinflammatory/profibrotic and calcification markers in peritoneal fluid or in tissue specimens taken during peritoneal membrane biopsy.

In conclusion, in our opinion aldosterone antagonist’s therapy is considered to be safe in PD patients and should be used more frequently. Further studies are necessary to evaluate their role in cardiovascular system protection and relationship with mineral-bone disturbances in PD pts.
